# Effects of Polyacrylic Acid Pre-Treatment on Bonded-Dentine Interfaces Created with a Modern Bioactive Resin-Modified Glass Ionomer Cement and Subjected to Cycling Mechanical Stress

**DOI:** 10.3390/ma11101884

**Published:** 2018-10-02

**Authors:** Salvatore Sauro, Vicente Faus-Matoses, Irina Makeeva, Juan Manuel Nuñez Martí, Raquel Gonzalez Martínez, José Antonio García Bautista, Vicente Faus-Llácer

**Affiliations:** 1 Departamento de Odontologia, Facultad de Sciencia de la Salud, Universidad CEU Cardenal Herrera, 46115 Valencia, Spain; juan.nunez@uchceu.es (J.M.N.M.); raquel.gonzalez@uchceu.es (R.G.M.); joseanto@uchceu.es (J.A.G.B.); 2 Department of Therapeutic Dentistry, Sechenov University Russia, 119435 Moscow, Russia; irina_markovina@mail.ru; 3 Departamento de Estomatología, Facultad de Medicina y Odontología, Universitat de Valencia, 46010 Valencia, Spain; fausvj@uv.es (V.F.M.); Vicente.J.Faus@uv.es (V.F.L.)

**Keywords:** adhesion, bioactive, cycling mechanical stress, dentine, longevity, resin-modified glass ionomer cements, polyacrylic acid treatment

## Abstract

**Objectives:** Resin-modified glass ionomer cements (RMGIC) are considered excellent restorative materials with unique therapeutic and anti-cariogenic activity. However, concerns exist regarding the use of polyacrylic acid as a dentine conditioner as it may influence the bonding performance of RMGIC. The aim of this study was to evaluate the effect of different protocols for cycling mechanical stress on the bond durability and interfacial ultramorphology of a modern RMGIC applied to dentine pre-treated with/without polyacrylic acid conditioner (PAA). **Methods:** The RMGIC was applied onto human dentine specimens prepared with silicon-carbide (SiC) abrasive paper with or without the use of a PAA conditioner. The specimens were immersed in deionised water for 24 h then divided in 3 groups. The first group was cut into matchsticks (cross-sectional area of 0.9 mm^2^) and tested immediately for microtensile bond strength (MTBS). The second was first subjected to load cycling (250,000 cycles; 3 Hz; 70 N) and then cut into matchsticks and tested for MTBS. The third group was subjected to load cycling (250,000 cycles; 3 Hz; 70 N), cut into matchsticks, and then immersed for 8 months storage in artificial saliva (AS); these were finally tested for MTBS. The results were analysed statistically using two-way ANOVA and the Student–Newman–Keuls test (α = 0.05). Fractographic analysis was performed using FE-SEM, while further RMCGIC-bonded dentine specimens were aged as previously described and used for interfacial ultramorphology characterisation (dye nanoleakage) using confocal microscopy. **Results:** The RMGIC applied onto dentine that received no pre-treatment (10% PAA gel) showed no significant reduction in MTBS after load cycling followed by 8 months of storage in AS (*p* > 0.05). The RMGIC–dentine interface created in PAA-conditioned SiC-abraded dentine specimens showed no sign of degradation, but with porosities within the bonding interface both after load cycling and after 8 months of storage in AS. Conversely, the RMGIC–dentine interface of the specimens with no PAA pre-treatment showed no sign of porosity within the interface after any of the aging protocols, although some bonded-dentine interfaces presented cohesive cracks within the cement after prolonged AS storage. However, the specimens of this group showed no significant reduction in bond strength (*p* < 0.05) after 8 months of storage in AS or load cycling (*p* > 0.05). After prolonged AS storage, the bond strength value attained in RMGIC–dentine specimens created in PAA pre-treated dentine were significantly higher than those observed in the specimens created with no PAA pre-treatment in dentine. **Conclusions:** PAA conditioning of dentine prior to application of RMGIC induces no substantial effect on the bond strength after short-term storage, but its use may increase the risk of collagen degradation at the bonding interface after prolonged aging. Modern RMGIC applied without PAA dentine pre-treatment may have greater therapeutic synergy with saliva during cycle occlusal load, thereby enhancing the remineralisation and protection of the bonding interface.

## 1. Introduction

Glass ionomer cements (GICs) were introduced for the first time in dentistry by Wilson and Kent in 1969 [1] as an innovative class of dental material able to set via an acid-base reaction after mixing fluoro-aluminosilicate glass particles (FAS) with a polyacrylic acid solution (PAA) [2]. Low viscosity polyacids, such as maleic and itaconic acids were incorporated within the PAA solution to improve the handling and setting of GICs [3,4,5]. Tartaric acid was also incorporated into the PAA solution to enhance the handling properties and increase the working time [6,7].

It is well known that the initial setting occurs due to a gelation reaction between the fluoro-aluminosilicate glass particles and polyalkenoate acids [8], followed by a proper hardening phase characterised by the cross-linking of the carboxylic groups present in the polymeric chains with calcium and aluminium ions present in the FAS. However, the final chemical reaction for complete setting occurs during the following 48 h [6,7], although the final “maturation” of the cement may take several months due to the slow release of aluminium ions from the glass particles. It is also important to highlight that sodium and fluoride ions are not usually involved in the setting reaction, but rather, these ions remain unreacted within the matrix, and are released gradually into the surrounding environment (e.g., bioactivity) [9,10]. Indeed, for this reason, GICs present unique therapeutic anti-cariogenic activity, which is mainly attributed to the release of fluoride (F^−^) ions and to their buffering properties [11,12,13,14].

Glass ionomer cements are being used for a wide range of applications in dentistry [2]. These include the restoration of deciduous teeth [15], anterior class III and V restorations [16,17], cementation (luting) of crowns, bridges and orthodontic appliances [18,19,20], restorations of non-carious teeth with minimal preparation [21,22], and sandwich technique restorations [23,24]. Furthermore, they comprise the main material for atraumatic restorative therapy (ART) [25]. Indeed, subsequent to selective removal of the caries-infected tissues, GICs are applied as therapeutic ion-releasing materials to remineralise the caries-affected tissue left behind inside the dental cavity [26,27]. GICs may also exhibit a number of drawbacks, such as brittleness [28], poor wear resistance, inadequate surface properties [29,30], and sensitivity to high moisture in the oral cavity when newly placed [31].

In order to overcome such drawbacks, several modifications have been introduced to conventional GICs [32,33,34]. A key modification was the reinforcement of GICs through the incorporation of urethane monomers to produce resin-modified glass ionomer cements (RMGICs) [35,36]. Unlike conventional GICs, RMGICs can be self-activated (self-polymerisation) or light-cured (photo-polymerisation reaction). These “hybrid” materials have been generated to combine the mechanical properties of resin monomers with the anti-carious potential of GICs [37]. Indeed, it has been observed that RMGICs not only release fluoride, but they may also have greater flexural strength and lower solubility compared to conventional GICs [36,37]. RMGICs have a decreased fluoride release and higher creep relative to conventional powder-based ionomers [7]. Although first generation of RMGICs presented slight expansion due to water sorption (from 3.4% up to 11.3%) 24 h after placement [38], modern formulations have overcome this problem [36]. Conventional RMGICs are also characterised by lower mechanical (e.g., Young’s modulus and flexural strength) and “inferior” aesthetic properties compared to resin composites [7,38].

A key advance of glass ionomer-based materials is their self-adhesive properties to bind chemically to calcium ions (Ca^2+^) in the apatite of enamel and dentine through chelation of carboxyl group of acidic polymeric chains [39,40,41]. However, the self-adhesive mechanism of GIC-based materials to dentine is also due to the micromechanical interlocking achieved by shallow hybridization of the micro-porous collagen network. A 10% solution of PAA is mostly used as an enamel/dentine conditioner to remove the smear layer prior to the application of GIC-based restorative materials. Nevertheless, concerns exist regarding its use, application time, and concentration, as these factors may interfere with the overall bonding performance. Indeed, a high number of adhesive failures between a RMGIC and resin composite have been reported when a polyalkenoic conditioner was used on smear-layer covered dentine [40,41]. Cycling occlusal stress occurring during mastication, swallowing, as well as in cases of parafunctional habits, can affect the integrity of the bonding interface, making it more susceptible to short and long term degradation in the oral environment [42].

The aim of this study was to evaluate the microtensile bond strength (MTBS), after short-term load-cycle aging or after load cycle followed by prolonged aging (8 months) in artificial saliva (AS), of a modern bioactive RMGIC applied to dentine with or without surface pre-conditioning using 10% polyacrylic acid (PAA). Fractographic analysis and interfacial dye-assisted nanoleakage assessment of the bonded interfaces were evaluated using field-emission scanning electron microscopy (FE-SEM) and confocal laser-scanning microscopy (CLSM), respectively.

The tested null hypotheses were that the durability of RMGIC applied with or without the use of a PAA conditioner would be affected by: (i) short-term load-cycle aging; (ii) or load cycle followed by prolonged aging (8 months) in AS.

## 2. Materials and Methods

### 2.1. Preparation of Dentine Specimens

Sound human molars were extracted for periodontal or orthodontic reasons and stored in distilled water at 5 °C for no longer than 3 months. The roots were removed 1 mm beneath the cemento–enamel junction using a diamond-embedded blade (high concentration XL 12205; Benetec, London, UK) mounted on a low speed microtome (Remet evolution, REMET, Bologna, Italy). A second parallel cut was made to remove the occlusal enamel and expose mid-coronal dentine.

Two main groups (n = 30 specimens/group) were created based on dentine pre-treatment. Group 1: Specimens were abraded using 320-grit SiC abrasive paper (1 min) under continuous irrigation, followed by a water rinse (20 s), and air-drying (3 s); they were then restored with a light-cured RMGIC (no PAA conditioning). Group 2: Specimens were abraded with 320-grit SiC abrasive paper (1 min), conditioned with 10% PAA gel for 20 s rinsed with water (20 s), dried (3 s), and restored with a light-cured RMGIC (PAA conditioning).

The restorative procedure was performed using the content of two mono-dose capsules of a commercial RMGIC (RIVA light cure HV, Bayswater, VIC, Australia), mixed for 10 s in a trituration unit, and applied in bulk on to the dentine surface and light-cured for 30 s with a light-curing unit (Radii plus, SDI Ltd, Bayswater VIC, Australia) with a LED light source (>1000 mW/cm^2^).

The experimental design required that each main group be subsequently subdivided into three sub-groups (n = 10 specimens) based on the aging protocol: (1) CRT: no aging (control, 24 h in deionised water); (2) LC: Load cycling (250,000 cycles in artificial saliva); (3) LC-AS: Load cycling (250,000 cycles in artificial saliva), followed by prolonged water storage (8 months in artificial saliva).

The composition of the artificial saliva was (AS: 0.103 g·L^−1^ of CaCl_2_, 0.019 g·L^−1^ of MgCl_2_·6H_2_O, 0.544 g·L^−1^ of KH_2_PO_4_, 30 g·L^−1^ of KCl and 4.77 g·L^−1^ HEPES (acid) buffer, pH 7.4). The specimens in the subgroup LC and LC-AS were mounted in plastic rings with acrylic resin for load cycle testing (250,000 cycles; 3 Hz; 70 N). A compressive load was applied to the flat surface of the RMGIC using a 5-mm diameter spherical stainless steel plunger attached to a cyclic loading machine (model S-MMT-250NB; Shimadzu, Tokyo, Japan) while immersed in AS [43].

### 2.2. Micro-Tensile Bond Strength (MTBS) and Fracture Analysis (FE-SEM)

The specimens were sectioned using a hard-tissue microtome (Remet evolution, REMET, Bologna, Italy) in both the X and Y planes across the dentine-RMGIC interface, obtaining approx. 20 matchstick-shaped specimens from each tooth with cross-sectional areas of 0.9 mm^2^. All the specimens were stored at 100% humidity, and were then (i) immediately cut into matchsticks, or (ii) load cycled and then cut into matchsticks, or (iii) load cycled, cut into matchsticks, and then stored for 8 months in AS; specimens were finally subjected to an MTBS test. The latter was performed using a microtensile bond strength device with a stroke length of 50 mm, peak force of 500 N, and a displacement resolution of 0.5 mm. Modes of failure were classified as a percentage of adhesive (A), mixed (M) or cohesive (C) failures when the failed interfaces were examined at 30X magnification by stereoscopic microscopy. Five representative fractured specimens from each sub-group were critical-point dried and mounted on aluminium stubs with carbon cement. The specimens were gold-sputter-coated and imaged using field-emission scanning electron microscopy (FE-SEM S-4100; Hitachi, Wokingham, UK) at 10 kV and a working distance of 15 mm.

Bond strength values in MPa were initially assessed for normality distribution and variances homogeneity using Kolmogorov-Smirnov and Levene’s tests, respectively. To analyse if the substrate pre-treatment approaches had an influence on the bond strength, two-way analysis of variance (pre-treatment and substrate condition) was performed. Chi-square analysis was performed to compare the results of failure mode between groups. The significance level was set at *p* ≤ 0.05. SPSS V16 for Windows (SPSS Inc., Chicago, IL, USA) was used.

### 2.3. Ultramorphology of the Bonded-Dentine Interfaces: Confocal Microscopy Evaluation

One dentine-bonded matchstick sample (Ø 0.9 mm^2^) was selected from the centre of each tooth in every experimental sub-group. These were coated with a fast-setting nail varnish, applied 1 mm from the bonded interface. They were immersed in a Rhodamine B (Merck KGaA, Darmstadt, Germany) water solution (0.1 wt.%) for 24 h. Subsequently, the specimens were ultrasonicated in distilled water for 5 min and then polished for 30 s each side with a 2400-grit SiC paper. The specimens were finally ultrasonicated again in distilled water for 5 min and submitted for confocal microscopy analysis. Using a confocal scanning microscope (Olympus FV1000, Olympus Corp., Tokyo, Japan) equipped with a 63X/1.4 NA oil-immersion lens and a 543 nm LED illumination, reflection and fluorescence images were obtained with a 1-µm z-step to optically section the specimens to a depth of up to 20 µm below the surface. The z-axis scan of the interface surface was pseudo-coloured arbitrarily for improved exposure and compiled into both single and topographic projections using the CLSM image-processing software (Fluoview Viewer, Olympus Corp., Tokyo, Japan). The configuration of the system was standardised and used at constant settings for the entire investigation [43]. Each dentine interface was investigated completely, and then five optical images were randomly captured. Micrographs representing the most common morphological features observed along the bonded interfaces were captured and recorded.

## 3. Results

### 3.1. Micro-Tensile Bond Strength (MTBS) and Failure Mode Analysis

Microtensile bond strength means and standard deviation are expressed in MPa in Table 1. Dentine surface treatments and aging in AS (8 months) had no significant influence on the MTBS results (*p* > 0.01). Interactions between factors were not significant (*p* > 0.05). The MTBS performed at 24 h with the non-load-cycled specimens showed that the use of PAA dentine conditioning induced no significant increase in bond strength (*p* > 0.05), compared to the specimens created by applying the RMGIC on smear layer-covered dentine (no PAA-treatment).

Likewise, after load cycling, the specimens created in dentine pre-treated using PAA showed a bond strength comparable (*p* > 0.05) to that obtained with the specimens created with the RMGIC applied onto dentine surfaces that received no PAA conditioning.

The specimens created in dentine pre-treated with PAA and those without PAA conditioning showed no significant difference (*p* > 0.05) after LC-AS aging compared to the specimens in the control group (24 h) or those in the group where the specimens where subjected to load cycling only. The only significant difference (*p* < 0.05) in terms of bond strength was observed after LC-AS aging; this occurred between the specimens created in dentine pre-treated with PAA and those without PAA conditioning.

### 3.2. Failure and Fractographic FE-SEM Analysis

Most of the specimens from all groups failed predominantly in cohesive mode within RMGIC (range: 70–88%) and in mixed mode (10–25%) after 24 h and load cycling aging (Table 1), while most of the specimens tested after LC-AS aging failed prevalently in mixed mode (range: 55–65%) compared to those tested after 24 h or load cycling only (*p* < 0.05). The percentage of adhesive failures after LC-AS aging was significantly higher (*p* < 0.05) (range: 10–13%) in the specimens in both PAA and no- PAA groups compared to those tested after 24 h or load cycling (range 2–5%).

**Figure 1:**
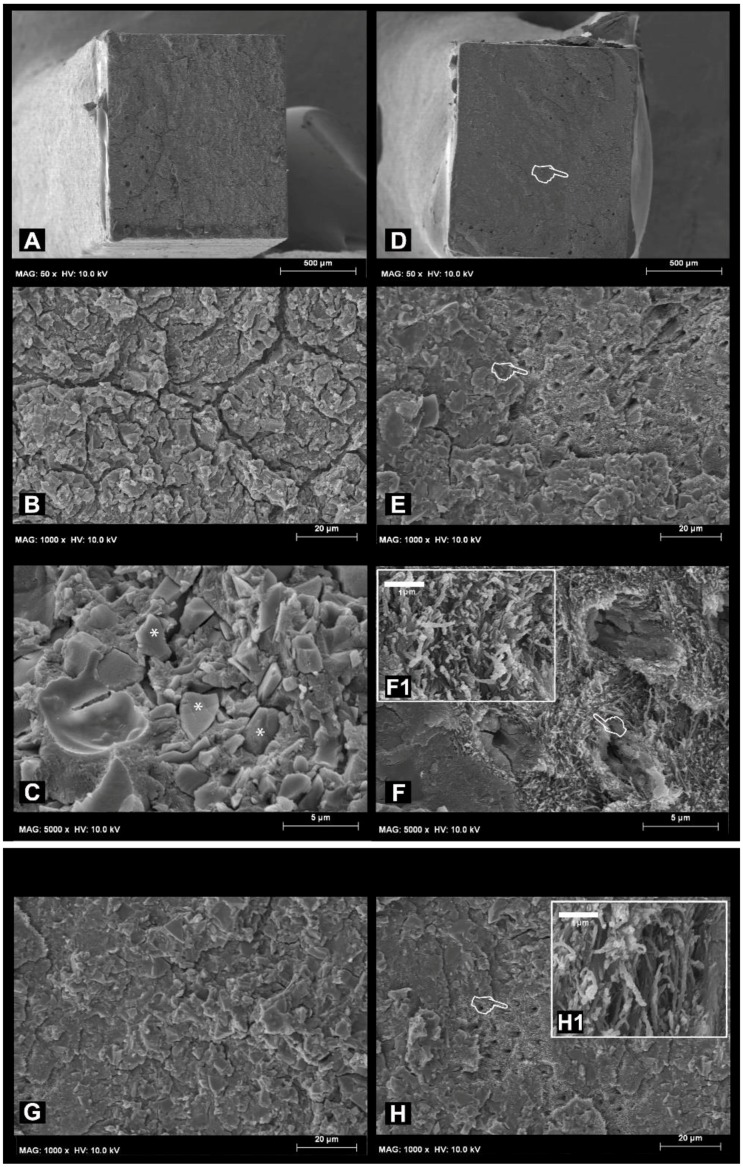
(**A**) Representative SEM micrograph of a specimen created with the use of no PAA applied on dentine that failed during the microtensile bong strength test in cohesive mode after 24 h of storage in water. At higher magnification, it is possible to note a surface covered by residual RMGIC (**B**) characterized by the presence of particles (*) of fluoroaluminosilicate glass (**C**). (**D**) Representative SEM fractographic analysis of specimens created with the use of PAA applied on dentine that failed in mixed mode after 24 h storage. At higher magnification, it is possible to see the presence of totally exposed unprotected dentinal tubules (pointer), and the presence of some partially demineralised residual collagen fibrils (pointer) (**E**,**F**,**F**-1). (**G**) Representative SEM micrograph of specimen created with the use of no PAA applied on dentine that failed during microtensile bond strength test in cohesive mode after load cycling aging. Also, in this case it is possible to note a surface covered by RMGIC with no dentine exposure. (**H**): Representative SEM fractographic analysis of specimens created with the use of PAA applied on dentine that failed in mixed mode after load cycling aging. It is possible to see the presence of totally exposed unprotected dentinal tubules (pointer), and, at a higher magnification, it is possible to note the presence of partially demineralised collagen fibrils (**H**-1).

**Figure 2:**
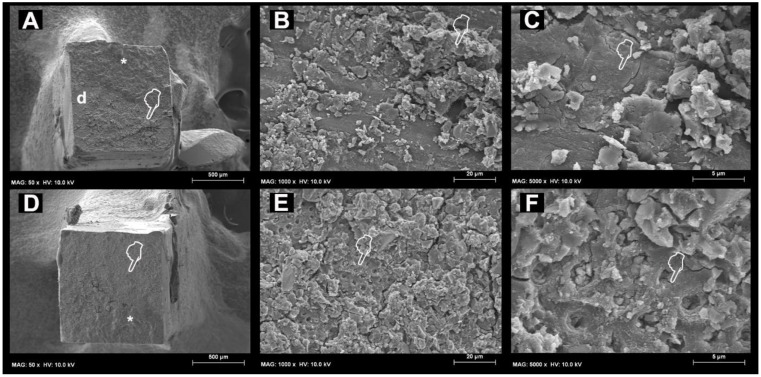
SEM micrographs obtained after load cycle followed by prolonged AS storage. (**A**) Representative SEM micrograph of specimen created with the use of no PAA applied on dentine that failed during microtensile bong strength test in mixed mode, where it is possible to see residual RMGIC (pointer), compact residual RMGIC (*) and some exposed dentine (d). (**B**) At higher magnification, it is possible to note a surface covered by residual RMGIC [pointer] and a dentine surface (pointer) with no exposed tubules but still covered by smear layer (**C**), (**D**); Representative SEM fractographic analysis of specimens created with the use of PAA applied on dentine that failed in mixed mode after 8 months of storage in AS which is characterised by the presence of residual RMGIC (*) and dentine (pointer). (**E**) At higher magnification, it is possible to observe the presence of exposed dentinal tubules (pointer) surrounded by residual RMGIC particles. (**F**) At even higher magnification it is possible to note the tubules are still occluded, but with no presence of exposed collagen fibrils, which probably degraded over time during prolonged aging in AS.

The SEM fractographic results at 24 h and after load cycling aging are shown in Figure 1. In short, the specimens created without PAA pre-treatment that failed during a microtensile bong strength test mainly in cohesive mode after 24 h of storage in water (Figure 1A) showed a surface covered by residual RMGIC (Figure 2B,C). The specimens created with the use of PAA re-treatment applied on dentine that failed in mixed mode after 24 h storage showed some areas with the presence of unprotected dentinal tubules, which were totally exposed and characterised by the presence of partially demineralised collagen fibrils (Figure 1F,1-F1). Also, the specimens created with the use of no PAA and then subjected to load cycling prevalently showed a surface covered by RMGIC with no exposure of the dentinal tubules (Figure 1G). The specimens created with the use of PAA applied on dentine showed after load cycling only the presence of totally exposed unprotected dentinal tubules (Figure 1F); at higher magnification, it was possible to observe the presence of partially-demineralised collagen fibrils (Figure 1H-1).

The SEM fractographic results after LC-AS aging are depicted in Figure 2. A residual presence of RMGIC (Figure 2B) and a dentine surface devoid of exposed tubules was observed in the specimens created with the use of no PAA mode (Figure 2A); the dentine surface devoid of exposed tubules was still covered by smear layer (Figure 2C). On the other hand, the specimens created with the use of PAA applied on dentine that failed in mixed mode after load cycling and 8 months of storage in AS were characterised by the presence of residual RMGIC and some exposed dentine (Figure 2D). At higher magnification, tubules which were still occluded by residual RMGI were detected, but with no presence of exposed collagen fibrils was observed; these probably degraded over time during the prolonged aging in AS.

### 3.3. Ultramorphology of the Bonded-Dentine Interfaces: Confocal Microscopy Evaluation

**Figure 3:**
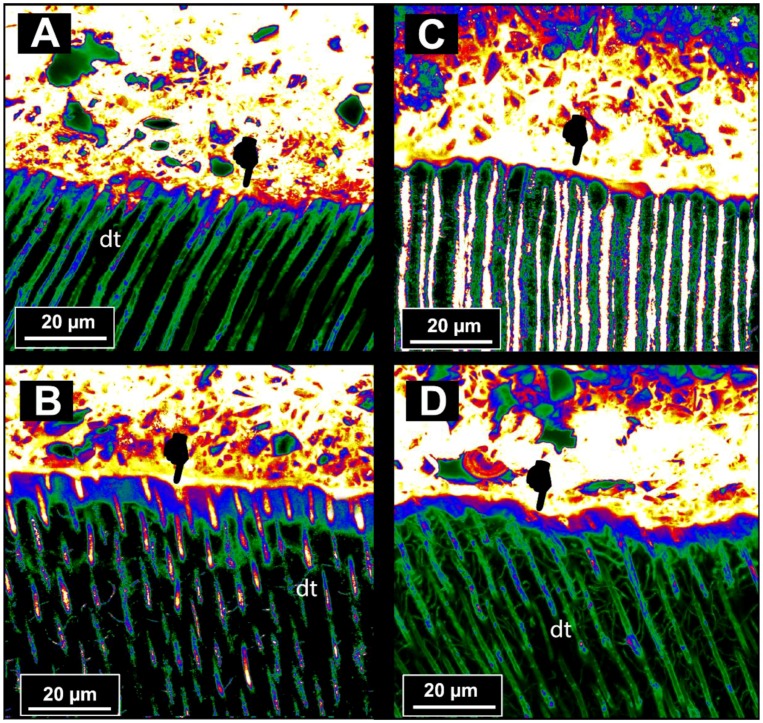
Confocal images of interfaces at 24 h or after short-term cycle load aging. (**A**) CLSM projection image exemplifies the interfacial characteristics at 24 h of the bond–dentine interface created by application of the resin-modified glass ionomer cement (RMGIC) onto dentine without PAA pre-treatment. It is possible to see a permeable gap-free interface that absorbed the fluorescein solution through the dentinal tubules (dt). In particular, this highlighted the existence of a thin interdiffusion layer (pointer). (**B**) CLSM projection at 24 of a representative bond-dentine interfaces created by RMGIC applied onto dentine pre-treated with PAA. In this case, it is possible to appreciate a thicker interdiffusion layer that absorbed the fluorescein solution through the dentinal tubules (dt) (**C**) A representative CLSM projection of a RMGIC-dentine interface created by applying the RMGIC onto a dentine pre-treated with no PAA and subjected to load cycling. It is possible to observe that such an aging protocol had no effect on the overall morphology of the interface, although the interdiffusion layer appears slightly thinner (pointer) than that observed in picture (**A**). (**D**) A representative CLSM projection of a RMGIC-dentine interface created by applying the RMGIC onto a dentine pre-treated with 10% PAA and subjected to load cycling. Also, in this case, it is possible to observe that such an aging protocol had no effect on the overall morphology of the interface, but the interdiffusion layer appears clearly thinner (pointer) than that observed in picture (**B**), which represents the same specimens subjected to no load-cycle aging.

The results of the ultramorphology and nanoleakage analysis of the RMGIC-dentine interfaces performed through dye-assisted confocal microscopy at 24 h and after load cycling only are shown in Figure 3. In short, at 24 h, the RMGIC applied onto dentine without PAA pre-treatment presented a gap-free interface characterised by a thin interdiffusion layer, which absorbed the fluorescent solution (Rhodamine B) through the dentinal tubules (Figure 3A). Conversely, the RMGIC applied onto the dentine pre-treated with PAA presented a thicker and more porous interdiffusion layer (Figure 3B). The RMGIC-dentine specimens created by applying the RMGIC onto the dentine pre-treated with no PAA and subjected to short-term load-cycle, showed an interdiffusion layer which was slightly thinner compared to that of the control specimens (24 h), (Figure 3C). This morphological features were also observed in the specimens bonded using RMGIC onto dentine pre-treated with 10% PAA and then subjected to load cycling. Indeed, such an aging protocol had no effect on the overall morphology of the interface, but the interdiffusion layer clearly appeared thinner than that observed in the specimens at 24 h (Figure 3C).

**Figure 4:**
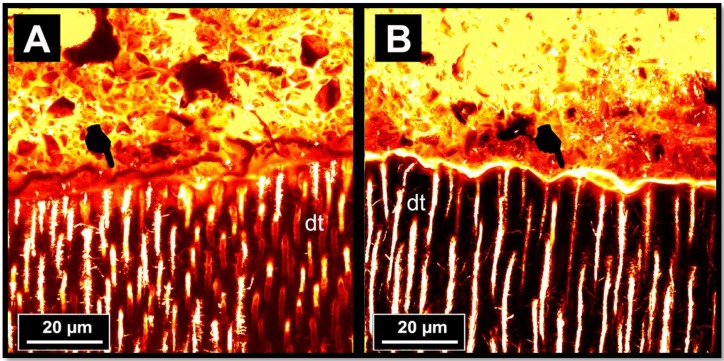
Confocal images of interfaces after cycle load aging and prolonged AS aging. (**A**) A representative CLSM projection of a RMGIC-dentine interface created by applying the RMGIC onto a dentine pre-treated with no PAA and subjected to prolonged AS storage. It is possible to observe the presence of a cohesive fracture within the RMGIC layer (pointer), probably created during specimen preparation (polishing) due to the brittle characteristics of such a material. This observation is supported by the absence of a permeable interfusion layer at RMGIC-dentine interface due to the maturation of the latter after prolonged storage in AS. Conversely, the RMGIC-dentine interface created by applying the RMGIC onto a dentine pre-treated with PAA and then subjected to prolonged AS storage (**B**) shows a remaining thin permeable interdiffusion layer (pointer), which indicates the presence of porosities created subsequent to collagen degradation during prolonged water storage.

The results of the ultramorphology and nanoleakage analysis of the RMGIC-dentine interfaces after LC-AS aging are shown in Figure 4. In this case, it was noted that the RMGIC-dentine interface of the RMGIC applied onto the dentine surface pre-treated with no PAA often presented cohesive fractures within the RMGIC layer; these were probably created during specimen preparation due to the brittle characteristics of such a material (Figure 4A). Conversely, the RMGIC-dentine interface created by applying the RMGIC onto a dentine pre-treated with PAA and then subjected to prolonged AS storage showed a remaining thin permeable interdiffusion layer, which may indicate the presence of porosities causing collagen degradation during subsequent prolonged water storage (Figure 4B).

## 4. Discussion

Therapeutic minimally invasive dentistry encompasses the philosophy of preservation of reparable dental tissues, along with the use of remineralising approaches to re-establish as much as possible of the mechanical properties of such tissues [44]. Glass-ionomer materials can be considered the main self-adhesives [45,46] and ion-releasing restorative materials available in clinics nowadays which are able to achieve such a target. However, it is believed that the overall bonding performance of such materials may be maximised if the dental substrates are pre-treated with a diluted polyacrylic acid conditioner (PAA 10%) [47,48]. Indeed, PAA conditioners remove the smear layer from dentine and enamel surfaces, consequently making the HAp directly accessible to interact with glass ionomer cements. Moreover, a slight dentine demineralisation occurs subsequent to PAA application, and a submicron interdiffusion layer is formed, which provides micromechanical retention [47,48,49]; the residual HAp within the demineralised collagen fibrils may also serve as receptors for additional chemical interaction [44,45,46,47,48]. The use of PAA as a dentine conditioner is still a theme of debate with modern resin-modified GIC [40,41], especially when considering its effect on the durability and remineralisation of dentine-bonded interfaces [43]. Furthermore, it has been recently demonstrated [50] that with conventional RMGICs such as Vitrebond Plus (3M ESPE), due to a great level energy accumulation at the dentine bonding interface during cycle load, there was evident fluorescent permeability associated with a lack of hermetic sealing.

The results of our study are in accordance those of Toledano at al. [50], as all the specimens applied in dentine pre-treated with or without PAA showed remining permeability at the bonding interface. Indeed, our current results demonstrated that the RMGIC tested in this study after the application on dentine without PAA pre-treatment presented a thin gap-free interface characterised by a thin layer of Rhodamine B absorbed through dentinal tubules (Figure 3A). In contrast, the interface of the RMGIC applied onto the dentine pre-treated with PAA was characterised by more Rhodamine B accumulation, which was due to a lack of sealing of dentinal tubules (Figure 3B), as well as a thicker layer of PAA-demineralised dentine, which remained non-infiltrated by the RMGIC [43,50].

However, after short-term load-cycle aging, the RMGIC-dentine specimens created using the RMGIC in dentine without PAA pre-treatment showed only a very thin interdiffusion layer characterised by slight fluorescence signal at the interface. As described by Toledano at al. [50], such a reduction in porosities at the bonding interface may have been due to apatite-like precipitation and remineralisation induced at the interface during mechanical cycling stress. Conversely, the specimens created with the RMGIC applied after PAA dentine pre-treatment also showed that the thickness fluorescent signal at the interface was reduced compared to the same specimens at 24 h (Figure 3C), although such a porous layer was thicker than that observed when using no PAA dentine pre-treatment. In this case, it is possible that the level of mineral precipitation was not so suitable for remineralising all the porosities within the interdiffusion layer, especially at its bottom. Indeed, Kim at al. [51] recently shown that GIC-based materials fail to completely remineralise apatite-depleted dentine due to a lack of nucleation of new apatite, even when biomimetic remineralising analogues were employed during the aging period [52].

It is important to highlight that such short-term load cycle aging was not able to induce any significant change in the microtensile bond strength, and no difference in the mode of failure in both groups of specimens (PAA vs. PAA dentine pre-treatment) was observed, compared to the control specimens at 24 h. Conversely, the results of this study showed that the specimens created with the representative RMGIC applied in dentine pre-treated with PAA showed a significant reduction in bond strength after LC-AS aging compared to specimens tested after 24 h or after short-term load cycle aging. Moreover, the LC-AS aging protocol induce also a significant change in the mode of failure; RMGIC applied in dentine pre-treated with or without PAA presented more adhesive qualities compared to all the other groups. Therefore, while the first null hypothesis is totally rejected, the second one that the durability of RMGIC applied with or without the use of PAA conditioner would be affected by load cycle followed by prolonged aging (8 months) in AS tested must be partially rejected.

Some of the current results are in accordance with those reported by Inoue et al. [45], who showed that the use of PAA conditioner before application of GIC-based materials offered no significant increase of the MTBS to dentine. Similar results were also recently reported by Sauro et al. [43], who showed that RMGIC applied onto dentine pre-treated with PAA showed significant μTBS reduction after 6 months of AS storage alone or in combination with load cycling (*p* > 0.05). Moreover, they also showed that for the RMGIC-dentine interface, specimens were affected by degradation/nanoleakage after aging, unlike the interfaces created without the use of PAA conditioning, which showed signs of remineralisation/maturation of the bonding interface.

The fractographic SEM analysis performed in this study showed that pre-treating the dentine with PAA could, in some cases, cause clear exposure of the collagen fibrils both before and after prolonged AS storage. Moreover, such fibrils were less abundant after prolonged AS storage compared to those observed in the specimens aged in AS for 24 h (Figure 2F). These specimens also showed a significant increase in the number of adhesive failures. In accordance with previously-published results [40,41,43], current fractographic SEM results showed that the specimens created with the use of no dentine PAA conditioner that failed in adhesive mode presented with dentine still covered by residual RMGIC, as the fracture occurred just above the dentine surface (Figure 2C).

It is hypothesised that such a result could be attributed to hydrolytic degradation processes that occurs over time within the collagen. Indeed, the use of PAA to pre-treat the dentine tissue may have demineralised the dentine collagen and activated endogenous matrix collagenolytic (MMP 1, MMP 8 and MMP 13) and gelatinolytic (MMP 2 and MMP 9) metalloproteinases [53]. It was also demonstrated [54] that high concentration of carboxylic groups in PAA acid conditioner may cause the formation a PAA-based polymeric gel layer within the bonding interface, which induces more water sorption at the interface. The RMGIC itself may also have degraded and become more porous over time in AS, thereby facilitating diffusion of water towards the glass-ionomer–dentine interface and causing an acceleration of the degradation processes [55].

The results of the ultramorphology and nanoleakage analysis of the RMGIC-dentine interfaces after prolonged AS storage showed that when RMGIC were applied onto the dentine surface, pre-treated with no PAA, and then subjected to prolonged AS storage, a thin permeable interdiffusion layer remained. This outcome may support the hypothesis that a bonding interface is usually characterised by the presence of porosities created subsequent to collagen degradation during prolonged water storage (Figure 4B). Conversely, the specimens created with the use of no PAA dentine conditioning showed that the absorbing layer at the interface seen at 24 h examination (Figure 3A) disappeared after prolonged AS storage (Figure 4A) due to the maturation of such areas [55], and possible remineralisation [43,56]. Indeed, the therapeutic properties (e.g., ion releasing) of RMGIC may have induced the growth of mineral crystals and remineralisation within the bonded-dentine interface, which interfered with the proteolytic action of endogenous dentine metalloproteinases [56,57]. This latter hypothesis is in accordance with previous studies that demonstrated the fluoride might inhibit both pro- and active forms of MMP 2 and MMP 9 [58]. Moreover, Makowski & Ramsby [59] reported that mineral precipitation, as well as apatite formation, may inhibit MMP activity through the formation of [Ca-PO/MMP] complexes. Sauro et al. [43] have recently reported that such a remineralising potential of RMGIC may increase if they are applied onto dentine pre-treated with bioactive glass in air-abrasion systems, and then conditioned with or without the use of a PAA conditioning gel.

In conclusion, within the limitations of this in vitro study, it is possible to affirm that the clinical decision of using a PAA conditioner should be based upon the histological features of the dentine retained after cavity preparation (e.g., sound or caries-affected dentine). However, modern RMGICs may be used for dentine restorations with or without the use of PAA pre-treatment, but it is important to consider that such a type of acid etching procedure might increase the risk of degradation at the bonding interface after prolonged service in oral cavity under mechanical cycling stress and prolonged saliva immersion. Conversely, in cases of no PAA dentine pre-treatment, it might be possible to have a synergic combination between GIC-based materials, saliva, and cycle occlusal load, which may enhance the therapeutic properties of RMGIC to induce mineralisation and protection of the bonding interface, thereby achieving more long-lasting restorations.

## Figures and Tables

**Table 1:** The results show the mean ± SD of the MTBS (MPa) to dentine when resin-modified glass ionomer cement was applied after different dentine pre-treatments.

Main Groups Dentine Etching (10% PAA gel)	24 h AS (CTR)	Load Cycling in AS (LC)	Load Cycling and 8-Month in AS (LC-AS)
No PAA (95/5)	16.3 ± 5.9 (A1)	16.4 ± 4.1 (A1)	13.1 ± 4.6 (A1)
	(5/25/70)	(2/10/88)	(10 */55 */35 *)
Yes PAA (100/0)	21.5 ± 4.8 (A1)	21.1 ± 5.5 (A1)	14.2 ± 5.2 (A2)
	(0/15/85)	(3/17/80)	(13 */65 */22 *)

Percentage (%) of total number of beams (intact sticks/pre-failed sticks) in the dentine treatment groups and percentage of failure modes (adhesive/mix/cohesive). The same letter indicates no differences in columns with different dentine treatments maintained in the same aging conditions. The same number indicates differences in rows for the same dentine treatment but different aging conditions (*p* > 0.05). The symbol (*) indicates significant differences in the mode of failure in the same treatment group after different aging conditions.
